# Vaccine Acceptance Among Pregnant Women in Israel During COVID-19: Influences and Decision-Making Factors

**DOI:** 10.3390/vaccines12121404

**Published:** 2024-12-12

**Authors:** Noa Feldman, Michal Bitan, Maya Alayev, Orna Tal

**Affiliations:** 1 The Shamir Medical Center (Assaf Harofeh), Rishon LeZion, 4 Icet, Zrifin 7033001, Israel; mayaa@shamir.gov.il (M.A.); ornatal@shamir.gov.il (O.T.); 2 School of Computer Science, The College of Management, Rishon LeZion 7570724, Israel; michalbi2@colman.ac.il; 3 Health Management Program, Faculty of Management, Bar Ilan University, Ramat Gan 5290002, Israel; 4 ICET, Israeli Center for Emerging Technologies, Ramat Gan 7033001, Israel; 5 Department of Health Sciences, Ramat Gan Academic College, Ramat Gan 5211401, Israel

**Keywords:** COVID-19, Influenza, Whooping Cough, T-dap, hesitancy, vaccine, attitudes, pregnant women

## Abstract

**Background and Objectives:** In response to the COVID-19 pandemic, Israel prioritized pregnant women for vaccination, recognizing them as a high-risk group. This study aims to explore factors influencing the acceptance of Influenza, Pertussis (T-dap), and COVID-19 vaccines among pregnant women, focusing on attitudes, social norms, perceived control, and risk perceptions. Additionally, the study compares acceptance patterns between traditional vaccines and the newer COVID-19 vaccine. **Methods:** A prospective cohort study was conducted between December 2019 and December 2021 involving 449 predominantly Israeli pregnant women. A survey was administered to gather data on demographics, obstetric history, vaccination history, and factors influencing vaccination decisions. **Results:** COVID-19 vaccine uptake was the highest at 64% (202/314), followed by T-dap at 49% (221/449) and Influenza at 32% (146/449). Multivariable logistic regression showed that non-religious women and those with academic education were more likely to accept vaccines, especially the COVID-19 vaccine. Physician recommendations were the most influential factor in vaccine acceptance, while internet and media sources played a significant role in shaping COVID-19 vaccine decisions. Perceived risks varied: Whooping Cough was seen as the greatest threat to newborns, while COVID-19 posed the highest risk to mothers. Analyzing maternal and neonatal disease perception using multivariable linear regression, we found that information on maternal and neonatal Flu, Whooping Cough, and COVID-19 was significantly positively correlated with disease perception for each condition. **Conclusions:** Healthcare providers play a crucial role in influencing vaccine decisions, especially through personalized communication. Strategies targeting religious communities and leveraging media can help address vaccine hesitancy, ultimately improving maternal and neonatal health outcomes.

## 1. Introduction

Healthcare providers worldwide aim to achieve high vaccination coverage, especially among vulnerable groups like children, the elderly, those with chronic conditions, and pregnant women. Israel has established a comprehensive vaccination policy with high compliance in these population [1], although vaccine hesitancy remains a challenge. Factors influencing hesitancy include knowledge about the disease and vaccine, trust in healthcare providers and authorities, personal health beliefs, and cultural and social influences [2]. Individual factors such as education, health literacy, and perceived risk also play a role. Globally, hesitancy is a multifaceted issue, with reasons for vaccine refusal including perceived risk–benefit concerns, religious beliefs, and knowledge gaps [3,4,5]. Many studies have documented maternal acceptance, vaccine perceptions, and provider knowledge about vaccines like Pertussis and Influenza [6,7,8,9,10], which are relevant to COVID-19 vaccine hesitancy as well. As of March 2021, Israel had administered 116 COVID-19 vaccine doses per 100 people, outpacing other OECD nations [11]. Key factors in Israel’s success included a centralized government structure, a well-coordinated healthcare system, quick funding, early vaccine procurement, prioritization, efficient cold storage, and effective outreach [11]. However, vaccine hesitancy persisted. Studies indicated a strong correlation between vaccine intent and perceived safety, even among healthcare workers [12], with higher hesitancy among religious groups [13,14] and those with negative attitudes toward the vaccine [15]. Young adults often cited low perceived COVID-19 risk and concerns about long-term side effects, particularly fertility risks among young women. Hesitancy was also fueled by rapid vaccine rollout and mRNA technology concerns, along with uncertainty about vaccine efficacy against new variants [11]. Pregnant women are particularly vulnerable to infectious diseases, including COVID-19. Vaccination during pregnancy provides protection for the mother, fetus, and newborn through passive immunity, where maternal antibodies are transferred to the fetus via the placenta [16]. This mechanism is most effective with certain immunoglobulins, primarily IgG [17]. The timing of vaccination during pregnancy impacts the level of antibodies transferred to the newborn, offering protection in the early months of life [17,18]. Vaccinations during pregnancy are categorized into routine immunizations, specific medical indications, and vaccines to be avoided and are provided free of charge by nurses at public community clinics [18].

Pertussis, which causes Whooping Cough, poses severe risks to infants under six months [19]. The U.S. CDC has recommended T-dap vaccination during pregnancy since 2011 [20], and Israel included it in routine prenatal care as of 2015 [18,21]. Studies confirm the safety and effectiveness of maternal T-dap vaccination, especially between 27 and 36 weeks of pregnancy, for optimal antibody transfer [22,23,24,25]. However, in Israel, Pertussis remains prevalent, with a notable susceptibility gap in infants younger than eight weeks, when immunization begins [21].

Influenza, a seasonal virus causing respiratory illness, is associated with severe complications in pregnant women due to physiological changes during pregnancy that increase susceptibility. The WHO reports that Influenza causes millions of severe cases and hundreds of thousands of deaths each year [26]. Pregnant women face increased risks, including secondary pneumonia, respiratory failure, preterm delivery, and fetal death, which make them a priority group for Influenza vaccination [27,28]. Studies link antenatal Flu vaccination with reduced risks of stillbirth, preterm delivery, and low birth weight in infants [29,30]. Since the 1960s, Flu vaccination has been a standard recommendation to protect both mother and child [28]. In Israel, the Influenza vaccine is part of the recommended immunization schedule for pregnant women. However, pregnant individuals should not receive live attenuated vaccines during pregnancy [18]. COVID-19 emerged as a global threat in early 2020, with millions of cases and fatalities reported worldwide [31]. Pregnant individuals with symptomatic COVID-19 are at higher risk for severe illness, ICU admission, and adverse pregnancy outcomes, including preterm birth and pre-eclampsia [32,33]. Though in utero transmission is rare and neonatal outcomes are generally favorable [34,35], the development of COVID-19 vaccines marked a major milestone in reducing risks for pregnant women. By the end of 2020, several COVID-19 vaccines were available, with widespread administration following WHO validation [31]. Vaccination reduces the risk of developing COVID-19 and reduces the severity of disease if a breakthrough infection occurs [36]. Initial clinical trials excluded pregnant women [37], creating early uncertainty about vaccine safety in this group; patients and their doctors initially were required to make decisions about vaccination. However, international health organizations later recommended COVID-19 vaccination for those planning pregnancies, those who are pregnant, and lactating mothers, as evidence grew regarding safety and efficacy [38,39,40,41]. Vaccination is shown to reduce disease severity in cases of breakthrough infection and offers passive immunity to the newborn. Concerns about potential risks, such as miscarriage or birth complications, have been largely dispelled by emerging data, which show no increased risks associated with COVID-19 vaccination during pregnancy [33,42]. In Israel, COVID-19 vaccination began in December 2020, with prioritized groups including older adults, nursing home residents, healthcare workers, and high-risk individuals. By April 2021, Israel had administered over 10 million doses, achieving high coverage (70%), particularly in adults aged 65 and over [11]. COVID-19 vaccination has since been integrated into routine annual vaccination schedules for healthcare workers and at-risk populations [41,43].

Vaccine hesitancy is a multifaceted issue influenced by psychological, social, cognitive, and cultural factors. The success of vaccination campaigns relies on public willingness to participate, which is shaped by factors such as disease and vaccine knowledge, perceived risks and benefits, trust in healthcare authorities, sociodemographic influences, and social media impact. This study seeks to assess the knowledge, attitudes, and willingness of Israeli pregnant women to receive vaccines for Influenza, Pertussis, and COVID-19, using the Theory of Planned Behavior (TPB) and Health Belief Model (HBM) as guiding frameworks [44,45]. To date, no similar study has been conducted in Israel. Given the limited research on maternal perceptions and acceptance of COVID-19 vaccines compared to established “traditional” vaccines, it is crucial to explore how attitudes toward these vaccines differ. Insights from this research could guide targeted strategies to improve vaccine acceptance among pregnant women.

## 2. Materials and Methods

### 2.1. Theoretical Framework

This study investigates the intention of pregnant women to receive the Influenza, Pertussis, and COVID-19 vaccines using the Theory of Planned Behavior (TPB) and the Health Belief Model (HBM) [44,45] as theoretical frameworks.

#### 2.1.1. Theory of Planned Behavior (TPB)

The TPB posits that a person’s intention to perform a specific behavior is the strongest predictor of the actual behavior. In the context of this study, we aim to understand how the following factors influence a pregnant woman’s intention to receive the vaccines:

- Attitude: Favorability towards receiving the vaccines;
- Subjective Norm: Perception of social pressure to get vaccinated (descriptive and injunctive norms);
- Perceived Behavioral Control: Belief in her ability to overcome barriers and get vaccinated [44].

#### 2.1.2. Health Belief Model (HBM)

The HBM focuses on risk perception as a key factor influencing health behaviors [44,45]. We will explore how the following influence a woman’s intention to receive the vaccines:

- Perceived Susceptibility: Belief about her chance of contracting Flu, Whooping Cough, and COVID-19
- Perceived Severity: Perception of the seriousness of Flu, Whooping Cough and COVID-19.

#### 2.1.3. Additional Considerations

Beyond the core constructs of the TPB and HBM, as mentioned, the study also considered the influence of

- Outcome Expectancies: Beliefs about the benefits and barriers associated with receiving the vaccines (e.g., vaccine effectiveness, safety concerns).
- Affective Factors: Anticipated regret about getting vaccinated or not getting vaccinated, fear of the different vaccines: Influenza, Whooping Cough, and COVID-19.
- Trust in Healthcare Institutions: Trust in the National Immunization Program and public health institutions.
- Influence of social media.
- Past Experiences: Past experiences with vaccinations.

### 2.2. Study Design

A prospective cohort study was conducted at the Fetal-Maternal outpatient clinic at Shamir Medical Center in Rishon Lezion, Israel. This public hospital, with 900 beds, serves as a central tertiary referral center specializing in high-risk pregnancies and neonatal intensive care. A convenience sample of pregnant women receiving antenatal care at the clinic was surveyed. Eligible participants, aged 18 and older, were identified through the daily clinic schedule and invited to participate. After reviewing an informational cover letter, consenting participants completed an anonymous written survey in Hebrew, with simultaneous translations available in Arabic, English, or Russian. The study was conducted in accordance with the Declaration of Helsinki, and approved by the Shamir Medical Center Institutional Review Board (ASF 0317-21).

### 2.3. Data Collection

A multidisciplinary team of maternity care providers and researchers designed the survey after a thorough literature review on vaccine acceptance during pregnancy, drawing on parameters from prior studies on vaccine attitudes [8,9,10]. The survey, conducted between 2019 and December 2021, included 29 questions using checkboxes and Likert scales to collect data on demographics, obstetric history, vaccine information sources, awareness of current recommendations, vaccination history, intent to vaccinate, and factors influencing vaccination decisions. Participants rated information, awareness, and barriers to vaccination on a 10-point scale, with 1 indicating “not at all” and 10 indicating “very strongly”. See Appendix A for the questionnaire.

### 2.4. Data Analysis

The data were analyzed using R Statistical Software (v4.4.0; R Core Team, Vienna, Austria, 2021). Descriptive statistics, including means, standard deviations, frequencies, and percentages, were used to summarize the demographic characteristics of the sample. Univariate and multivariable logistic regression models were performed to identify factors associated with maternal vaccination decisions for each disease. To evaluate the influence of various sources (physician, nurse, article, friend, internet, media, self) on vaccination decisions across COVID-19, Influenza, and Pertussis, a mixed model analysis of variance with Holm’s correction for multiple comparisons was conducted. Ratings of each factor were treated as repeated measures, with vaccination type as the independent variable. Interaction plots were generated to show significant subgroup differences. Additionally, two mixed-model ANOVAs with interaction plots were used to assess differences in maternal perceptions of disease severity for mother and fetus and vaccination uptake rates across the three vaccination types. A *p*-value below 0.05 was considered statistically significant.

## 3. Results

Out of the 500 consecutive pregnant women invited to participate in the study, 449 women (89.8%) completed the survey, between December 2019 and December 2021. As of January 2021, we added questions regarding the COVID-19 vaccine, and of the 449, only 314 completed the updated questionnaire. Each pregnant woman’s participation was distinct and limited to a single instance during the study period. Table 1 displays the demographic and vaccination date of the pregnant women.

The average age of the participants was 32.1 years. Most participants were married (89%), had an academic background (61%), were born in Israel (80%), and identified as Jewish (87%). Regarding religiosity, 79% identified as secular, or light-orthodox (traditional), 15% as orthodox, and 5.9% as Ultra-Orthodox. The mean parity was 1.64 (ranging from 0 to 7). The mean gestational age was 29 weeks. Seventeen percent of participants did not have children prior to the study. Among participants with children, 56% reported vaccinating their child, while 36.7% did not vaccinate their children. Seventy-six percent of participants received at least one vaccine.

Among those who received only one vaccine (35%), the COVID-19 vaccine had the highest uptake at 17%. When focusing solely on the population that completed the questionnaire, including COVID-19-related data (314 participants), this rate rose to 23.8%. In contrast, the Influenza vaccine had the lowest uptake at 5.6%, while the T-dap vaccine uptake alone was 13%. Overall, 202 out of 314 participants (64%) received the COVID-19 vaccine, followed by 221 out of 449 (49%) who received the T-dap vaccine and 146 out of 449 (32%) who received the Influenza vaccine. To delineate the "profile" indicating willingness to accept vaccinations, logistic regression analysis was conducted to explore correlations between various factors. This analysis began by exploring the correlation between individual characteristics and vaccination status. Table 2 presents the statistical analysis of the factors that influenced the coverage of the tested vaccines. Among the characteristics examined, after adjusting for other variables, non-religiosity was consistently associated with vaccination across all three vaccines (*p* 0.001–0.013). Additionally, individuals with academic education levels were significantly more likely to receive the COVID-19 vaccine, with an odds ratio of 2.2 (95% CI: 1.33–3.65). In contrast, higher education was not a significant factor in Influenza or Pertussis vaccination.

Jewish individuals were significantly more likely than non-Jewish individuals to receive both the COVID-19 and Pertussis vaccines. Additionally, each additional week of pregnancy was associated with a 1.33 times higher likelihood of receiving the T-dap vaccine (95% CI: 1.20–1.48), indicating a growing inclination for Whooping Cough vaccination as pregnancy advances, although this trend was not observed for COVID-19 or Influenza. This finding held true even when examining changes from 27 to 36 weeks’ gestation (*p* = 0.001).

**Figure 1:**
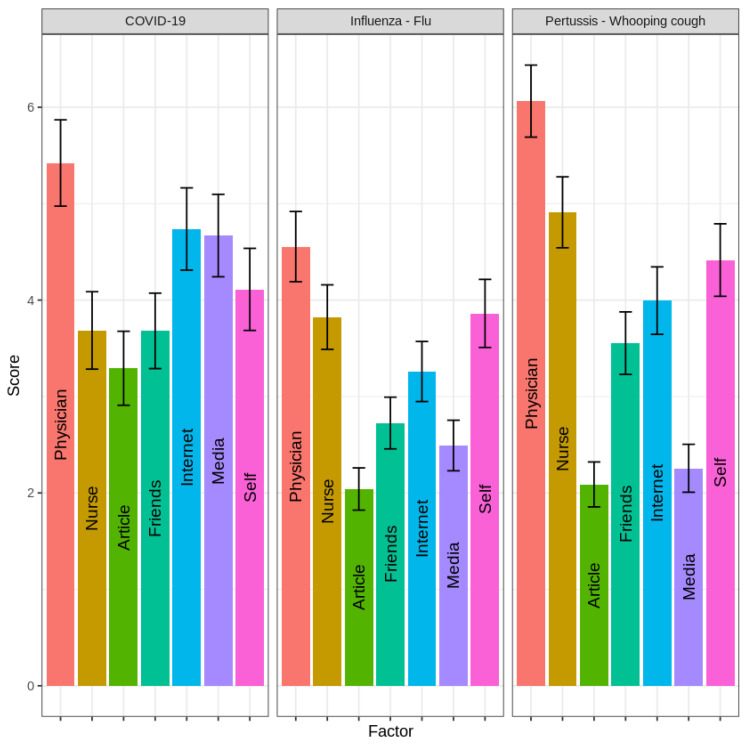
Comparison of the relative impact of professional (doctors, nurses, medical articles) and informal (internet, friends, media, personal experiences) sources on vaccination decisions.

We also assessed the impact of various sources of information, including professional sources (doctor’s recommendations, nurse’s recommendations, and medical articles) and informal sources (internet, friends, media, and personal experiences). The influence of each source on vaccination rates was classified as low, medium, or high, as illustrated in Figure 1.

Using a mixed model analysis of variance with Holm’s correction for multiple comparisons, the physician’s recommendation emerged as the most influential factor across all three vaccines, although the impact of other sources varied by vaccine.

For the COVID-19 vaccine, there was no significant difference between the influence of the physician’s recommendation and that of the internet and media (*p-adj* > 0.05). The nurse’s recommendation, personal experience, friends’ advice, and article influence had similar effects, with no significant difference among these factors, though each parameter differed significantly from the first group (*p-adj* < 0.001).

For the T-dap vaccine, the physician’s recommendation was the strongest factor in vaccination decisions (*p-adj* < 0.001), followed by the nurse’s recommendation and personal experience, with no significant difference between the two (*p-adj* > 0.05). The least influential sources, grouped together (*p-adj* < 0.001), included friends’ advice, media, the internet, and articles. For the Influenza vaccine, four distinct influence levels emerged: the physician’s recommendation was the most influential (*p-adj* < 0.001), followed by the nurse’s recommendation, personal experience, and the internet, which had similar effects but differed significantly from other groups (*p-adj* < 0.001). Friends and media formed the third influence group, distinct from the other categories (*p-adj* < 0.001), with articles having the least influence.

**Figure 2:**
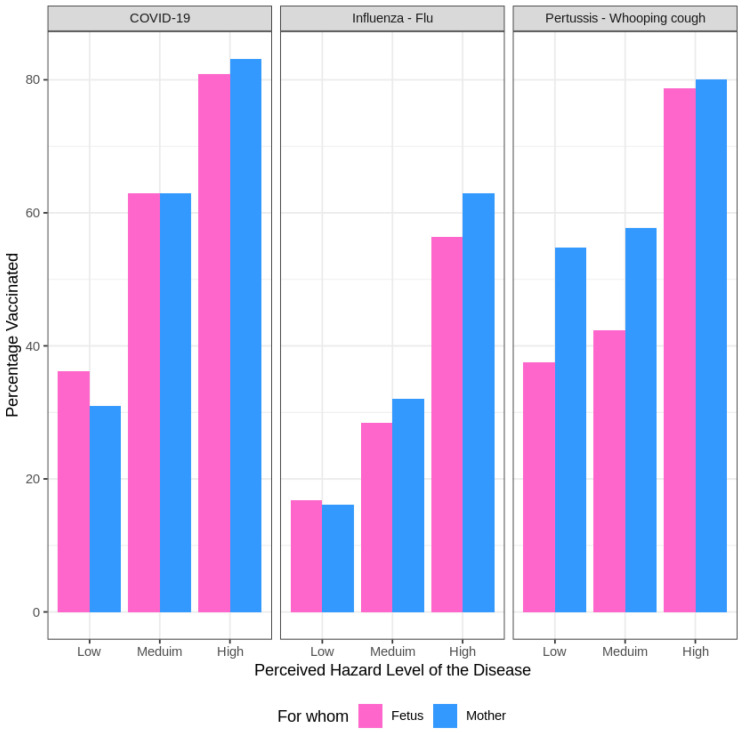
Impact of perceived disease risk to mother and fetus/newborn on vaccine acceptance across three vaccine types.

Our investigation delved into the variations in perception concerning the potential risks posed by the three diseases to both the mother and the fetus across the three vaccines. Figure 2 depicts the willingness to receive vaccination in correlation with the perception of the potential risks posed by the diseases to both the mother and the fetus or newborn across the three vaccines.

There is a statistically significant trend ( p<0.001), where pregnant women often perceive the diseases as carrying a greater risk for their fetus or newborn compared to themselves. Notably, Whooping Cough appears to be generating the most concern for neonatal safety, while COVID-19 is perceived as posing a higher risk to the mother. Additionally, Whooping Cough is also seen as a threat to maternal health. In contrast, Influenza is viewed as the least dangerous disease for both the mother and fetus.

Analyzing maternal disease perception using multivariable linear regression, we found that information on maternal Flu, Whooping Cough, and COVID-19 was significantly positively correlated with disease perception for each condition (all p<0.001, Table 3). Similarly, fetal/neonatal disease perception was significantly associated with information on each disease (all p<0.001, Table 3).

Similarly, fetal/neonatal disease perception was significantly associated with information on each disease (all p<0.001, Table 4). Additionally, disease-specific information correlated positively with vaccine uptake for Influenza, Whooping Cough, and COVID-19 ( p=0.008). These findings indicate that increased awareness is strongly linked to both disease perception and vaccination uptake.

Using multivariable linear regression (Table 3) to analyze the impact of personal characteristics on disease risk perception, we found that religious affiliation significantly correlated with lower perceived risks for COVID-19 and Influenza compared to non-religious individuals. However, this association was not observed for Whooping Cough. Additionally, advanced gestational age and higher education levels were associated with reduced perceived risk of maternal Whooping Cough, with gestational week and academic background showing a negative correlation with this perception ( p=0.025). These findings suggest that personal and demographic factors shape how pregnant women evaluate risks associated with different diseases.

Regarding perceived neonatal risk (Table 4), religiosity was negatively associated with Whooping Cough risk perception ( p=0.009). Specifically, non-orthodox mothers perceived a higher neonatal risk compared to their orthodox and Ultra-Orthodox counterparts, suggesting that orthodox and Ultra-Orthodox mothers view the risk of Whooping Cough for neonates as lower. In examining associations between the established vaccines and COVID-19, we identified a significant positive bidirectional correlation ( p=0.009,r=0.21) between the likelihood of choosing to vaccinate (or not) for Flu and the decision to vaccinate (or not) for COVID-19 during pregnancy.

## 4. Discussion

Antenatal vaccination against Influenza, Pertussis, and COVID-19 not only safeguards mothers from these illnesses but also serves as a crucial measure in protecting infants during their initial three months of life. In Israel, the Ministry of Health, along with organizations like the Israeli Boards of Pediatrics and Obstetrics and Gynecology, has made significant efforts to stress the importance of Influenza and T-dap vaccination during pregnancy [18]. During the COVID-19 pandemic, coordinated efforts led to the vaccination of 70% of the general population [46] and contributed to the notable COVID-19 vaccination rate of 64% among pregnant women in our cohort. The COVID-19 pandemic has further emphasized the need to vaccinate pregnant women, further highlighting the imperative nature of maternal vaccination initiatives [38,39,40].

Our study offers valuable new insights into the attitudes of pregnant women, for whom the questions regarding the COVID-19 vaccine were not theoretical, as the decisions to receive the vaccine during pregnancy were imminent and impactful. The findings highlight new, key factors influencing vaccine acceptance within the Israeli population, including the role of religiosity, the influence of the internet and social media, and the predominantly passive approach to acquiring vaccination knowledge.

Vaccine hesitancy is not a new phenomenon; it dates back to the earliest vaccination efforts. In 17th-century England, objections to vaccines arose from concerns about safety, compounded by resistance to compulsory vaccination laws during the Victorian era, reflecting broader social and political tensions [47].

One important observation in our population is the significant role of religiosity in women’s decision-making about vaccination during pregnancy. Non-religious women consistently perceived a higher risk from diseases (COVID-19, Influenza for the mother; Whooping Cough for the neonate), which may account for their higher vaccination rates across all three vaccines. This suggests that religiosity may also impact acceptance levels for each vaccine differently.

Differences in vaccine hesitancy between religious and non-religious groups may reflect a stronger sense of autonomy and confidence in navigating social pressures among women from secular backgrounds. Limited access to health resources might also contribute to these differences. Vaccine hesitancy among religious individuals is not unique to Israel; in various settings, some religious leaders have discouraged vaccination, viewing it as conflicting with natural or moral principles [48]. In Israel, some orthodox communities tend to have lower vaccination rates, although comprehensive research on this trend is limited [49]. In Israel, certain orthodox communities exhibit lower vaccination rates, though comprehensive research on this trend remains scarce [49]. For instance, during the 2018–2019 national measles outbreak, the Tel Aviv District reported a significant surge in cases, with 413 patients affected. Among them, 100 (24%) were infants under one-year-old, and 230 cases (56%) occurred within the Ultra-Orthodox Jewish community. This group was disproportionately impacted due to factors such as dense living conditions, high birth rates, and frequent exposure to crowded religious settings. This outbreak underscores the critical need to address immunity gaps and ensure optimal healthcare planning to prevent future outbreaks [50]. Unfortunately, the outbreak within the Orthodox community in Israel extended globally, contributing to a related outbreak within the Orthodox community in New York [51]. Our findings align with a recent study from the School of Public Health at Tel Aviv University, which found that among Jewish participants, those identifying as orthodox or mildly orthodox were 20% less likely to receive the COVID-19 vaccine than non-religious participants [52]. These differences in vaccine hesitancy between religious and non-religious groups underscore the need for tailored education and communication strategies to address the concerns of religious communities. Additionally, higher education levels were significantly associated with an increased likelihood of receiving the COVID-19 vaccine, especially among non-religious and highly educated women. Women who opted for vaccination reported receiving more information about the disease than those who chose not to, suggesting that education may help overcome social barriers and dispel misconceptions about vaccination. Notably, non-Jewish women were less likely than Jewish women to receive the COVID-19 vaccine, which may stem from minority status and related distrust of government authorities. This finding aligns with similar results from the general population in Israel [11].

Looking into the differences between the three vaccines, we noticed that T-dap vaccination rates were higher than those for Influenza but lower than COVID-19 vaccination rates, reflecting varying misconceptions about these vaccines. Our findings suggest that women perceive COVID-19 and Whooping Cough as significant risks to both mother and newborn. Concerns about maternal Whooping Cough were negatively correlated with academic status and positively associated with religiosity, particularly among Orthodox women. This pattern likely reflects misconceptions about maternal risk, which appear more prevalent in Orthodox communities. In our cohort, T-dap uptake reached 49%, notably lower than the 80% reported in a previous study on postpartum women in northern Israel [53]. While we cannot fully explain this disparity, it is worth noting that other studies worldwide report vaccination rates more consistent with our findings, such as 58% and 43% [54,55]. Despite both being considered “traditional” vaccines, Influenza vaccination rates were lower than T-dap. This trend, observed beyond our population, may stem from misunderstandings about the risks of maternal Influenza [55]. In analyzing the associations between Influenza and COVID-19 vaccination, we identified a significant positive bidirectional correlation between the likelihood of choosing to vaccinate (or not) for Influenza and the decision to vaccinate (or not) for COVID-19 during pregnancy. Despite this correlation, Influenza vaccine uptake was considerably lower than COVID-19 vaccine uptake (32% versus 64%). This disparity may stem from persistent misconceptions about Influenza and the efficacy or necessity of the Influenza vaccine.

Healthcare providers should utilize real-time data on vaccine efficacy and safety to address misconceptions regarding Whooping Cough and Flu, emphasizing their proven benefits in protecting both mother and baby. The increase in T-dap acceptance later in pregnancy highlights a growing sense of responsibility for the newborn. Providers can leverage this period to educate expectant mothers about the vaccine’s importance in safeguarding their baby’s health.

In modern times, vaccine hesitancy often stems from concerns about vaccine safety and efficacy and distrust in pharmaceutical companies or government agencies. Despite robust scientific evidence supporting vaccination, misinformation amplified by social media continues to influence public opinion. Using a framework categorizing information sources into professional, social, and self-directed channels, our study provided valuable insights into how pregnant women acquire vaccine-related information. This approach enabled a comprehensive analysis of how different information sources impact vaccine decisions. Comparing the acceptance of established vaccines with newer ones further highlighted differences in acceptance patterns, offering insights to improve vaccine uptake among pregnant women.

Several factors contribute to vaccine acceptance, including a sense of responsibility to protect oneself, future generations, and the community. Trust in health authorities, personal autonomy in health decisions, and the ability to weigh risks and benefits all influence this decision-making process. Knowledge acquisition is crucial and can be active or passive, with a strong preference for evidence-based information.

Our findings indicate strong support for immunization among pregnant women, especially when recommended by healthcare providers, primarily physicians. This underscores the influential role of doctors, who hold a higher professional status in Israel, as well as the trust placed in health authorities, in shaping vaccination decisions. Vaccine knowledge acquisition was largely passive, with limited self-directed information-seeking reported (6% for Influenza, 7.8% for Pertussis, and 19.9% for COVID-19). For COVID-19, however, the influence of media and the internet was as strong as physician recommendations, likely due to the extensive coverage during the early pandemic. In contrast, neonatal Pertussis and Influenza receive limited media attention. This disparity likely contributed to the relatively high COVID-19 vaccination rate (64%), aligning with findings from studies on vaccine hesitancy factors in Israel and worldwide [11,49,56].

A recent review on COVID-19 vaccine hesitancy revealed rates ranging from 26% to 57%, attributing hesitancy to factors such as the rapid development and approval process and insufficient knowledge about the vaccine [56]. Similar to our findings, this review emphasized the importance of healthcare professionals’ trust and recommendations in improving vaccine uptake. It also highlighted that obtaining information from official sources was linked to positive vaccine attitudes, while reliance on non-scientific social media correlated with heightened anxiety about infection and vaccine risks. Interestingly, our study showed high trust in media and internet sources, with a strong correlation between higher information levels and increased vaccine acceptance. The perception of COVID-19 risk was closely tied to its pandemic nature, widespread impact, and the associated uncertainty. Health authorities must acknowledge the media’s powerful role in shaping public perceptions of disease risk and vaccine benefits. Additionally, personal experience proved to be a significant factor, showing a clear positive correlation between past vaccination history and future vaccination intentions. This suggests that prior experiences with vaccination enhance an individual’s ability to overcome barriers to immunization.

Israel was the first country to vaccinate its entire population, with a 70% success rate [11]. Even though initial vaccine trials excluded pregnant women, pregnant women were still vaccinated early on due to their high-risk status. Our findings indicate a high COVID-19 vaccination rate among pregnant women, likely influenced by doctors’ recommendations and media exposure, as shown by the strong correlation between perceived disease risk and vaccine uptake. Mothers who chose to vaccinate noted a higher risk from COVID-19 compared to those who did not vaccinate. Our study has several limitations. First, the sample size may limit the generalization of the findings to the broader population of pregnant women in Israel and worldwide. Second, the study focused on a cohort of high-risk pregnant patients, which may reduce its applicability to the general population. Third, the cohort primarily included Jewish women, reflecting Israel’s general population but limiting the representation of non-Jewish populations. Lastly, the pre-COVID-19 pandemic cohort consisted of only 135 participants, which restricts the ability to draw strong conclusions when comparing data between the two periods.

## 5. Conclusions

In conclusion, our study highlights the critical role of proactive communication by healthcare providers and media campaigns in addressing vaccine hesitancy. This combined strategy likely contributed to Israel’s success in vaccinating 70% of its population early in the pandemic as well as 49% of our cohort.

To enhance vaccination programs effectively, strategies by government and healthcare workers should include the following.

- Building Trust and Confidence
  – 1. Transparent Communication: learly convey vaccine safety, efficacy, and potential side effects.
  – 2. Countering Misinformation: Actively debunk anti-vaccine messages on social media with evidence-based information from trusted figures.
  – 3. Sharing Data: Publish large-scale studies demonstrating vaccines’ effectiveness in reducing infections, hospitalizations, and deaths to mitigate hesitancy.
  – 4. Media Collaboration: Partner with trusted media outlets to promote vaccination, especially for pregnant women, to enhance public trust.
- Addressing Concerns and Hesitancy
  – 1. Proactive Campaigns: Launch engaging, multi-platform communication campaigns highlighting vaccine safety and benefits.
  – 2. Educational Initiatives: Develop accessible, culturally specific materials to address misconceptions and encourage informed decision-making.
  – 3. Targeted Messaging: Address concerns about long-term fetal and fertility risks with evidence-based messaging and emphasize the consequences of maternal Flu, neonatal Whooping Cough, and COVID-19.
  – 4. Community-Specific Outreach:
    * Ultra-Orthodox Community: Use religious language and values, with trusted physicians and rabbinic leaders addressing fertility concerns.
    * Engaging Leaders: Establish task forces to collaborate with influential figures in Ultra-Orthodox and Israeli Arab communities to promote vaccination.
- Enhancing Accessibility Accessible Vaccines: Provide free vaccines to all residents, ensuring equitable access and reinforcing the government’s commitment to public health.
  By implementing these strategies, public understanding and acceptance of vaccines can improve, ensuring more successful vaccination programs.

## Figures and Tables

**Table 1:** Demographic and vaccination data of surveyed cohort of women. T-dap: Tetanus Diphtheria Pertussis.

Characteristic	Number Completing the Question	N = 449 ^1^
Influenza Vaccine	433	
No		264 (61%)
Intent to		23 (5.3%)
Yes		146 (34%)
T-dap Vaccine	436	
No		141 (32%)
Intent to		74 (17%)
Yes		221 (51%)
COVID-19 Vaccine	314	
No		100 (32%)
Intent to		12 (3.8%)
Yes		202 (64%)
Number_Vaccination	449	
1		109 (24%)
2		159 (35%)
3		133 (30%)
4		48 (11%)
Number of COVID-19 Vaccine Doses	268	
1		13 (4.9%)
2		67 (25%)
3		139 (52%)
I was vaccinated and recovering		49 (18%)
Type Vaccination	449	
All		48 (11%)
COVID-19 and Influenza		19 (4.2%)
Influenza and T-dap		54 (12%)
None		109 (24%)
Only COVID-19		75 (17%)
Only Influenza		25 (5.6%)
Only T-dap		59 (13%)
T-dap and COVID-19		60 (13%)
Age	442	
Mean (SD)		32.1 (5.3)
Min, Max		18.0, 51.0
Education	436	
Non-academic		171 (39%)
Academic		265 (61%)
Israeli	449	352 (78%)
Marital Status	443	
Not-Married		48 (11%)
Married		395 (89%)
Number Children	447	
Mean (SD)		1.42 (1.30)
Min, Max		0.00, 7.00
Religion	440	
Jewish		382 (87%)
Non-Jewish		58 (13%)
Religiosity	441	
Non-Orthodox (Secular) or Light-Orthodox		351 (80%)
Orthodox		64 (15%)
Ultra-Orthodox		26 (5.9%)
Gestational Age Week	439	
Mean (SD)		29 (7)
Min, Max		9, 39

^1^ n (%).

**Table 2:** Statistical analysis of factors influencing the uptake of the Influenza, T-dap, and COVID-19 vaccines.

	Univariable			Multivariable		
Characteristic	OR ^1^	95% CI ^1^	*p*-Value	OR ^1^	95% CI ^1^	*p*-Value
Influenza Vaccine						
**Gestational Age Week**	1.00	0.97, 1.02	0.742			
**Number Children**	0.86	0.74, 1.01	0.062			
**Age**	1.03	0.99, 1.07	0.162	1.02	0.98, 1.06	0.298
**Religiosity**			<0.001			<0.001
Non-Orthodox (Secular) or light-Orthodox	—	—		—	—	
Orthodox	0.43	0.23, 0.79		0.47	0.24, 0.86	
Ultra-Orthodox	0.12	0.02, 0.41		0.12	0.02, 0.42	
**Education**			0.045			0.094
Non-Academic	—	—		—	—	
Academic	1.51	1.01, 2.29		1.44	0.94, 2.22	
**Israeli**			0.615			
No	—	—				
Yes	0.89	0.56, 1.42				
**Religion**			0.395			
Jewish	—	—				
Non-Jewish	0.78	0.42, 1.38				
T-DAP Vaccine						
**Gestational Age Week**	1.10	1.07, 1.14	<0.001	1.10	1.07, 1.14	<0.001
**Number Children**	0.90	0.77, 1.05	0.192			
**Age**	1.02	0.98, 1.06	0.396			
**Religiosity**			0.015			0.017
Non-orthodox (secular) or light-orthodox	—	—		—	—	
Orthodox	0.53	0.30, 0.93		0.57	0.31, 1.04	
Ultra-Orthodox	0.40	0.17, 0.93		0.35	0.14, 0.83	
**Education**			0.710			
Non-academic	—	—				
Academic	1.08	0.71, 1.64				
**Israeli**			0.921			
No	—	—				
Yes	0.98	0.59, 1.58				
**Religion**			<0.001			
Jewish	—	—				
Not-Jewish	0.35	0.19, 0.62				
COVID-19 Vaccine						
**Gestational Age Week**	1.01	0.97, 1.04	0.652			
**Number Children**	1.05	0.88, 1.27	0.577			
**Age**	1.04	1.00, 1.09	0.054			
**Religiosity**			0.010			
Non-Orthodox (Secular) or Light-Orthodox	—	—				
Orthodox	0.44	0.23, 0.85				
Ultra-Orthodox	0.37	0.15, 0.95				
**Education**			0.001			0.001
Non-Academic	—	—		—	—	
Academic	2.27	1.38, 3.73		2.27	1.38, 3.73	
**Israeli**			0.204			
No	—	—				
Yes	1.45	0.81, 2.55				
**Religion**			0.022			
Jewish	—	—				
Non-Jewish	0.45	0.23, 0.89				

^1^ OR = Odds Ratio, CI = Confidence Interval.

**Table 3:** Statistical analysis of factors influencing maternal perceived risk of Influenza, T-dap, and COVID-19.

	Univariable				Multivariable		
Characteristic	N	Beta	95% CI ^1^	*p*-Value	Beta	95% CI ^1^	*p*-Value
Influenza Vaccine							
**Pregnancy Week**	404	−0.02	−0.06, 0.03	0.418			
**Number Children**	409	−0.19	−0.43, 0.05	0.113			
**Age**	407	−0.06	−0.12, 0.00	0.053			
**Religiosity**	406			0.003			0.009
Non-Orthodox (secular) or Light-Orthodox		—	—		—	—	
Orthodox		−0.91	−1.8, −0.05		−0.62	−1.4, 0.15	
Ultra-Orthodox		−1.9	−3.3, −0.62		−1.7	−2.9, −0.51	
**Education**	401			0.242			
Non-Academic		—	—				
Academic		-0.38	-1.0, 0.26				
**Israeli**	411			0.934			
No		—	—				
Yes		0.03	−0.72, 0.79				
**Information Flu**	402	0.43	0.35, 0.51	<0.001	0.42	0.34, 0.50	<0.001
T-DAP Vaccine							
**Pregnancy Week**	398	−0.02	−0.06, 0.03	0.514	−0.07	−0.11, −0.02	0.005
**Number Children**	403	−0.06	−0.31, 0.19	0.641			
**Age**	401	−0.07	−0.13, −0.01	0.027			
**Religiosity**	400			0.073			
Non-Orthodox (secular) or Light-Orthodox		—	—				
Orthodox		−0.73	−1.7, 0.19				
Ultra-Orthodox		−1.3	−2.8, 0.10				
**Education**	397			0.020			0.025
Non-Academic		—	—		—	—	
Academic		−0.81	−1.5, −0.13		−0.73	−1.4, −0.09	
**Israeli**	405			0.938			
No		—	—				
Yes		0.03	−0.78, 0.84				
**Information Whooping Cough**	396	0.39	0.30, 0.49	<0.001	0.42	0.32, 0.51	<0.001
COVID-19 Vaccine							
**Pregnancy Week**	289	0.00	−0.06, 0.05	0.909			
**Number Children**	291	0.07	−0.22, 0.35	0.639			
**Age**	291	0.04	−0.03, 0.11	0.271			
**Religiosity**	290			0.020			0.026
Non-Orthodox (secular) or Light-Orthodox		—	—		—	—	
Orthodox		−1.3	−2.3, −0.25		−1.3	−2.3, −0.32	
Ultra−Orthodox		−1.3	−2.8, 0.23		−0.86	−2.4, 0.64	
**Education**	287			0.052			
Non-academic		—	—				
Academic		0.78	−0.01, 1.6				
**Israeli**	293			0.608			
No		—	—				
Yes		0.24	−0.69, 1.2				
**Information COVID-19**	288	0.12	0.07, 0.17	<0.001	0.12	0.07, 0.18	<0.001

^1^ CI = Confidence Interval.

**Table 4:** Statistical Analysis of Factors Influencing Perceived Fetal Risk from Influenza, T-dap, and COVID-19.

	Univariable				Multivariable		
Characteristic	N	Beta	95% CI ^1^	*p*-Value	Beta	95% CI ^1^	*p*-Value
**Influenza** **Vaccine**							
**Pregnancy Week**	381	0.00	−0.04, 0.05	0.935			
**Number Children**	386	−0.11	−0.36, 0.14	0.378			
**Age**	384	0.00	−0.06, 0.06	0.913			
**Religiosity**	383			0.095			
Non-Orthodox (secular) or Light Orthodox		—	—				
Orthodox		−0.88	−1.8, 0.01				
Ultra-Orthodox		−0.87	−2.3, 0.59				
**Education**	381			0.576			
Non-Academic		—	—				
Academic		−0.19	−0.87, 0.48				
**Israeli**	388			0.840			
No		—	—				
Yes		−0.08	−0.87, 0.71				
**Information Flu**	383	0.32	0.23, 0.41	<0.001	0.32	0.23, 0.41	<0.001
**T-DAP Vaccine**							
**Pregnancy Week**	390	0.04	0.00, 0.08	0.048			
**Number Children**	395	−0.21	−0.43, 0.01	0.065			
**Age**	393	−0.01	−0.07, 0.04	0.655			
**Religiosity**	392			0.015			0.009
Non-Orthodox (secular) or Light-Orthodox		—	—		—	—	
Orthodox		−1.1	−1.9, −0.29		−0.94	−1.7, −0.20	
Ultra-Orthodox		−0.97	−2.3, 0.35		−1.3	−2.5, −0.06	
**Education**	389			0.736			
Non-academic		—	—				
Academic		0.10	−0.50, 0.71				
**Israeli**	397			0.404			0.018
No		—	—		—	—	
Yes		0.30	−0.41, 1.0		0.82	0.14, 1.5	
**Information Whooping Cough**	390	0.37	0.29, 0.45	<0.001	0.38	0.29, 0.46	<0.001
**COVID-19 Vaccine**							
**Pregnancy Week**	275	−0.02	−0.07, 0.03	0.453	−0.02	−0.08, 0.03	0.416
**Number Children**	277	0.06	−0.23, 0.35	0.682	0.13	−0.21, 0.47	0.442
**Age**	277	0.01	−0.06, 0.08	0.788	−0.01	−0.09, 0.07	0.843
**Religiosity**	276			0.337			0.267
Non-orthodox (secular) or Light Orthodox		—	—		—	—	
Orthodox		−0.52	−1.6, 0.52		−0.74	−1.9, 0.39	
Ultra-Orthodox		−0.97	−2.6, 0.61		−1.0	−2.6, 0.63	
**Education**	273			0.275			0.350
Non-Academic		—	—		—	—	
Academic		0.44	−0.36, 1.2		0.39	−0.43, 1.2	
**Israeli**	279			0.817			0.976
No		—	—		—	—	
Yes		−0.11	−1.0, 0.83		0.01	−0.96, 0.99	
**Information COVID-19**	277	0.09	0.04, 0.15	<0.001	0.09	0.04, 0.15	0.001

^1^ CI = Confidence Interval.

## Data Availability

The data are available from the corresponding author upon request

## References

[1] Turner D., Woloszko N., Chalaux T., Dek M. (2022). What Explains the Striking Differences in Vaccination Uptake Across OECD Countries?. https://oecdecoscope.blog/2022/04/12/what-explains-the-striking-differences-in-vaccination-uptake-across-oecd-countries/.

[2] Ramot S., Tal O. (2023). Supporting healthcare workers in vaccination efforts. Hum. Vaccines Immunother..

[3] Lane S., MacDonald N.E., Marti M., Dumolard L. (2018). Vaccine hesitancy around the globe: Analysis of three years of WHO/UNICEF Joint Reporting Form data-2015–2017. Vaccine.

[4] Wagner A.L., Masters N.B., Domek G.J., Mathew J.L., Sun X., Asturias E.J., Ren J., Huang Z., Contreras-Roldan I.L., Gebremeskel B. (2019). Comparisons of Vaccine Hesitancy across Five Low- and Middle-Income Countries. Vaccines.

[5] Child T.L., Health A. (2019). Vaccine hesitancy: A generation at risk. Lancet Child Adolesc. Health.

[6] Aharon A.A. (2011). Parents’ Decisions Not to Vaccinate Their Child: Past and Present, Characterization of the Phenomenon and Its Causes. Israeli Journal of Health Education and Promotion. www.health.gov.il/UnitsOffice/HD/PH/HealthEducation/Documents/4th/190_4th.pdf.

[7] Keller-Stanislawski B., Englund J.A., Kang G., Mangtani P., Neuzil K., Nohynek H., Pless R., Lambach P., Zuber P. (2014). Safety of immunization during pregnancy: A review of the evidence of selected inactivated and live attenuated vaccines. Vaccine.

[8] Anraad C., Lehmann B.A., Visser O., van Empelen P., Paulussen T.G., Ruiter R.A., Kamp L., van der Maas N.A., Barug D., Ruijs W.L. (2020). Social-psychological determinants of maternal Pertussis vaccination acceptance during pregnancy among women in the Netherlands. Vaccine.

[9] Dubé E., Gagnon D., Kaminsky K., Green C.R., Ouakki M., Bettinger J.A., Brousseau N., Castillo E., Crowcroft N.S., Driedger S.M. (2020). Vaccination during pregnancy: Canadian maternity care providers’ opinions and practices. Hum. Vaccines Immunother..

[10] Healy C.M., Rench M., Gandhi M., Perez C., Swaim L. (2014). Knowledge and Attitudes of Pregnant Women Towards Recommendations for Immunization During Pregnancy. Open Forum Infect. Dis..

[11] Rosen B., Waitzberg R., Israeli A., Hartal M., Davidovitch N. (2021). Addressing vaccine hesitancy and access barriers to achieve persistent progress in Israel’s COVID-19 vaccination program. Isr. J. Health Policy Res..

[12] Ramot S., Tal O. (2023). Attitudes of Healthcare Workers in Israel towards the Fourth Dose of COVID-19 Vaccine. Vaccines.

[13] Martens J.P., Rutjens B.T. (2022). Spirituality and religiosity contribute to ongoing COVID-19 vaccination rates: Comparing 195 regions around the world. Vaccine: X.

[14] Olagoke A.A., Olagoke O.O., Hughes A.M. (2020). Intention to Vaccinate Against the Novel 2019 Coronavirus Disease: The Role of Health Locus of Control and Religiosity. J. Relig. Health.

[15] Karlsson L.C., Soveri A., Lewandowsky S., Karlsson L., Karlsson H., Nolvi S., Karukivi M., Lindfelt M., Antfolk J. (2021). Fearing the disease or the vaccine: The case of COVID-19. Personal. Individ. Differ..

[16] Faucette A.N., Unger B.L., Gonik B., Chen K. (2015). Maternal vaccination: Moving the science forward. Hum. Reprod. Update.

[17] Eberhardt C.S., Blanchard-Rohner G., Lemaître B., Combescure C., Othenin-Girard V., Chilin A., Petre J., Martinez de Tejada B., Siegrist C.A. (2017). Pertussis Antibody Transfer to Preterm Neonates After Second- Versus Third-Trimester Maternal Immunization. Clin. Infect. Dis..

[18] (2018). Vaccines and Pregnancy. Israeli Position Paper Number 35. https://cdn.mednet.co.il/2019/09/35-חיסונים-והריון.pdf.

[19] Forsyth K., Plotkin S., Tan T., Wirsing von König C.H. (2015). Strategies to Decrease Pertussis Transmission to Infants. Pediatrics.

[20] Liang J.L., Tiwari T., Moro P., Messonnier N.E., Reingold A., Sawyer M., Clark T.A. (2018). Prevention of Pertussis, Tetanus, and Diphtheria with Vaccines in the United States: Recommendations of the Advisory Committee on Immunization Practices (ACIP). MMWR. Recomm. Rep..

[21] of Health Israel M. (2008). Increase in Pertussis Among Israeli Infants. https://www.health.gov.il/NewsAndEvents/SpokemanMesseges/Pages/07052013_2.aspx.

[22] Englund J. (2007). The Influence of Maternal Immunization on Infant Immune Responses. J. Comp. Pathol..

[23] Roberts J.N., Gruber M.F. (2015). Regulatory considerations in the clinical development of vaccines indicated for use during pregnancy. Vaccine.

[24] Kim D., Riley L., Harriman K., Hunter P., Bridges C. (2017). Advisory Committee on Immunization Practices Recommended Immunization Schedule for Adults Aged 19 Years or Older—United States, 2017. Am. J. Transplant..

[25] (2017). Committee Opinion No. 718: Update on Immunization and Pregnancy: Tetanus, Diphtheria, and Pertussis Vaccination. Obstet. Gynecol..

[26] Nair H., Brooks W.A., Katz M., Roca A., Berkley J.A., Madhi S.A., Simmerman J.M., Gordon A., Sato M., Howie S. (2011). Global burden of respiratory infections due to seasonal Influenza in young children: A systematic review and meta-analysis. Lancet.

[27] Grohskopf L.A., Blanton L.H., Ferdinands J.M., Chung J.R., Broder K.R., Talbot H.K., Morgan R.L., Fry A.M. (2022). Prevention and Control of Seasonal Influenza with Vaccines: Recommendations of the Advisory Committee on Immunization Practices — United States, 2022–23 Influenza Season. MMWR. Recomm. Rep..

[28] Roeca C., Polotsky A.J. (2017). Influenza vaccination in early pregnancy. Vaccine.

[29] Mosby L.G., Rasmussen S.A., Jamieson D.J. (2011). 2009 pandemic Influenza A (H1N1) in pregnancy: A systematic review of the literature. Am. J. Obstet. Gynecol..

[30] Prasad N., Huang Q.S., Wood T., Aminisani N., McArthur C., Baker M.G., Seeds R., Thompson M.G., Widdowson M.A., Newbern E.C. (2019). Influenza-Associated Outcomes Among Pregnant, Postpartum, and Nonpregnant Women of Reproductive Age. J. Infect. Dis..

[31] World Health Organization COVID-19 Weekly Epidemiological Update, Edition 120. https://www.who.int/publications/m/item/weekly-epidemiological-update-on-covid-19-30-november-2022.

[32] Seasely A.R., Blanchard C.T., Arora N., Battarbee A.N., Casey B.M., Dionne-Odom J., Leal S.M., Moates D.B., Sinkey R.G., Szychowski J.M. (2022). Maternal and Perinatal Outcomes Associated With the Omicron Variant of Severe Acute Respiratory Syndrome Coronavirus 2 (SARS-CoV-2) Infection. Obstet. Gynecol..

[33] Badell M.L., Dude C.M., Rasmussen S.A., Jamieson D.J. (2022). COVID-19 vaccination in pregnancy. BMJ.

[34] Jamieson D.J., Rasmussen S.A. (2022). An update on COVID-19 and pregnancy. Am. J. Obstet. Gynecol..

[35] Allotey J., Kew T., Fernández-García S., Gaetano-Gil A., Yap M., Sheikh J., Littmoden M., Akande O., Khalil H., Kumaran M. (2022). SARS-CoV-2 positivity in offspring and timing of mother-to-child transmission: Living systematic review and meta-analysis. BMJ.

[36] World Health Organization (2021). Coronavirus Disease (COVID-19) Vaccines Safety. https://www.who.int/news-room/questions-and-answers/item/coronavirus-disease-(covid-19)-vaccines-safety.

[37] PfizerBiontech (2020). COVID-19 Vaccine (BNT162, PF-07302048) Vaccines and Related Biological Products Advisory Committee briefing document. https://www.fda.gov/media/144246/download.

[38] Obsterics A.C. Gynecology. COVID-19 Vaccination Considerations for Obstetric-Gynecologic Care. https://www.acog.org/clinical/clinical-guidance/practice-advisory/articles/2020/12/covid-19-vaccination-considerations-for-obstetric-gynecologic-care.

[39] (2020). National Advisory Committee on Immunization (NACI) COVID-19 Vaccines: Canadian Immunization Guide—Pregnancy and Breastfeeding. https://www.canada.ca/en/public-health/services/publications/healthy-living/canadian-immunization-guide-part-4-active-vaccines/page-26-covid-19-vaccine.html#a6.1.

[40] UK Health Security Agency (2020). COVID-19 Vaccination: A Guide on Pregnancy and Breastfeeding. https://www.gov.uk/government/publications/covid-19-vaccination-women-of-childbearing-age-currently-pregnant-planning-a-pregnancy-or-breastfeeding/covid-19-vaccination-a-guide-on-pregnancy-and-breastfeeding.

[41] Obstetrics T.N.C., Gynecology N., Genetics (2012). COVID-19 Vaccination Women of Childbearing Age Currently Pregnant Planning a Pregnancyorbreastfeeding. https://cdn.mednet.co.il/2020/12/חיסון-נשים-הרות-עמדת-האיגוד-20.12.20.pdf.

[42] Rasmussen S.A., Kelley C.F., Horton J.P., Jamieson D.J. (2021). Coronavirus Disease 2019 (COVID-19) Vaccines and Pregnancy: What Obstetricians Need to Know. Obstet. Gynecol..

[43] HealthIM (2021). COVID-19 Reservoir. https://data.gov.il/dataset/covid-19.

[44] Ajzen I. (1991). The theory of planned behavior. Organ. Behav. Hum. Decis. Process..

[45] Janz N.K., Becker M.H. (1984). The Health Belief Model: A Decade Later. Health Educ. Q..

[46] Rosen B., Waitzberg R., Israeli A. (2021). Israel’s rapid rollout of vaccinations for COVID-19. Isr. J. Health Policy Res..

[47] Durban N. (2000). They might as well brand us: Working class resistance to compulsory vaccination in Victorian England. Soc. Soc. Hist. Med..

[48] Conner M.T., Norman P. (2005). Predicting Health Behaviour: Research and Practice with Social Cognition Models.

[49] Lyren A., Leonard E. (2006). Vaccine Refusal: Issues for the Primary Care Physician. Clin. Pediatr..

[50] Salama M., Indenbaum V., Nuss N., Savion M., Mor Z., Amitai Z., Yoabob I., Sheffer R. (2020). A Measles Outbreak in the Tel Aviv District, Israel, 2018–2019. Clin. Infect. Dis..

[51] Zucker J.R., Rosen J.B., Iwamoto M., Arciuolo R.J., Langdon-Embry M., Vora N.M., Rakeman J.L., Isaac B.M., Jean A., Asfaw M. (2020). Consequences of Undervaccination—Measles Outbreak, New York City, 2018–2019. New Engl. J. Med..

[52] Ber I., Lerman Y.M. (2021). K. The need for reducing disparities in sars-cov-2 immunization: The ultraorthodox and arab populations in israel. Harefuah.

[53] Bord S., Satran C., Madjar B. (2018). Vaccine compliance and hesitation among pregnant women in Israel. Eur. J. Public Health.

[54] van Zoonen K., Ruijs W.L.M., De Melker H.E., Bongers M.E.J., Mollema L. (2021). How to increase awareness of additional vaccinations; the case of maternal Pertussis vaccination. BMC Public Health.

[55] Doraivelu K., Boulet S.L., Biswas H.H., Adams J.C., Haddad L.B., Jamieson D.J. (2019). Predictors of tetanus, diphtheria, acellular Pertussis and Influenza vaccination during pregnancy among full-term deliveries in a medically underserved population. Vaccine.

[56] Gianfredi V., Stefanizzi P., Berti A., D’Amico M., De Lorenzo V., Lorenzo A.D., Moscara L., Castaldi S. (2023). A Systematic Review of Population-Based Studies Assessing Knowledge, Attitudes, Acceptance, and Hesitancy of Pregnant and Breastfeeding Women towards the COVID-19 Vaccine. Vaccines.

